# Bone remodeling induced by mechanical forces is regulated by miRNAs

**DOI:** 10.1042/BSR20180448

**Published:** 2018-07-03

**Authors:** Yue Wang, Lingfei Jia, Yunfei Zheng, Weiran Li

**Affiliations:** 1 Department of Orthodontics, Peking University School and Hospital of Stomatology, Beijing 100081, P.R. China; 2 Central Laboratory, Peking University School and Hospital of Stomatology, Beijing 100081, P.R. China; 3 Department of Oral and Maxillofacial Surgery, Peking University School and Hospital of Stomatology, Beijing 100081, P.R. China

**Keywords:** Bone remodeling, compression, fluid shear stress, mechanical cyclical stretch, microgravity, microRNA

## Abstract

The relationship between mechanical force and alveolar bone remodeling is an important issue in orthodontics because tooth movement is dependent on the response of bone tissue to the mechanical force induced by the appliances used. Mechanical cyclical stretch (MCS), fluid shear stress (FSS), compression, and microgravity play different roles in the cell differentiation and proliferation involved in bone remodeling. However, the underlying mechanisms are unclear, particularly the molecular pathways regulated by non-coding RNAs (ncRNAs) that play essential roles in bone remodeling. Amongst the various ncRNAs, miRNAs act as post-transcriptional regulators that inhibit the expression of their target genes. miRNAs are considered key regulators of many biologic processes including bone remodeling. Here, we review the role of miRNAs in mechanical force-induced bone metabolism.

## Introduction

Bone remodeling, which involves a cross-talk between osteoclasts and osteoblasts, is regulated by a number of proteins that interact through complex mechanisms [1]. Mechanical force can induce resident cell populations to adapt, maintain, and repair the bone structure. The *in vivo* milieu to which osteoblasts and osteoclasts are exposed is dynamic and changeable, and is where strain, stress, shear, pressure, fluid flow, streaming potential, and acceleration forces regulate bone remodeling [2]. Thus, studying the effects of mechanical forces on bone cells *in vitro* will improve our understanding of bone remodeling.

Mechanical forces play essential roles in bone remodeling. Mechanical cyclical stretching (MCS), fluid shear stress (FSS), compression, and microgravity play different roles in cell differentiation and proliferation by affecting intracellular interactions. Although the mechanisms are unclear, regulation by non-coding RNAs (ncRNAs) play an indispensable role in bone metabolism. ncRNAs, which are not translated into proteins [3], include miRNAs, siRNAs, piwi-interacting RNAs, small non-translated nucleolar RNAs (snoRNAs), small nRNAs, and long ncRNAs (lncRNAs). miRNAs, which are short ssRNAs of 18–25 nts, modulate orthodontic tooth movement (OTM) and alveolar bone remodeling in normal and inflammatory microenvironments *in vivo* [4,5]. LncRNAs are associated with the osteodifferentiation of human adipose-derived stem cells (ASCs) [6]. As part of the complex miRNA–mRNA–lncRNA regulatory network, lncRNAs influence bone formation and resorption in patients with osteoporosis [7]. Moreover, constitutive expression or silencing of lncRNA H19 is related to BMP9-induced osteogenic differentiation [8]. snoRNAs are also involved in bone formation. The snoRNA Snord116 is closely related to the bone-mass phenotype in people with Prader–Willi syndrome [9]. Circular RNAs (circRNAs) may play important roles in bone formation [10], and the circRNA–miRNA–mRNA network may function in osteogenesis [11].

Few studies have investigated mechanical force-induced changes in the expression of ncRNAs related to bone metabolism. Indeed, the effects of mechanical forces on lncRNA and snoRNA expression have been investigated less extensively than their effects on miRNAs. Hence, we reviewed the effects of mechanical force on miRNAs during bone remodeling.

## Effects of mechanical forces on bone metabolism

### MCS

MCS is involved in bone formation. Long-term mechanical stretching of human adipose tissue multipotential stromal cells (hAT-MSCs) leads to early osteogenesis [12], which has been confirmed *in vivo* [2,13–16]. Production of PDL-specific markers, including periostin and tenascin, can be stimulated by mechanical stress and can enhance cell proliferation [17]. The NGF-TrkA signaling pathway promotes communication between osteoblasts and sensory nerves in mice undergoing MCS [2]. The NOTCH and PERK-eIF2 α-ATF4 signaling pathways are also involved in osteodifferentiation [18,19]. These processes alter the expression levels of diverse osteogenesis-associated genes including osteopontin (*OPN*), osteocalcin, runt-related transcription factor 2 (*RUNX2*), and type I collagen [20,21].

### FSS

FSS promotes osteoblast proliferation [22] and induces Ca^2+^ influx via transient receptor potential cation channel subfamily V member 4 (TRPV4) channels and osteogenic differentiation of MSCs. These effects are inhibited by a selective TRPV4 blocker and TRPV4 siRNA [23]. FSS-induced up-regulation of cyclin B1 and CDK1 through the Gq-dependent ERK5 signaling pathway promotes the proliferation of MC3T3-E1 cells [22]. AMP-activated protein kinase signaling in BMSCs is involved in adiponectin-mediated prevention of FSS-induced cell death [24]. Moreover, the effects of FFS differ according to cell surface chemistry; osteoblasts have higher sensitivity to, and lower tolerance for, –OH and –CH_3_ surfaces compared with –NH_2_ surfaces [25]. FSS enhances and weakens calcium oscillations in osteoblasts in the early (4 days) and late (8 days) stages of induction, respectively [26]. The effects of FSS on osteoclasts are mediated by signaling pathways involving mechanosensitive and cation-selective channels, phospholipase C, and the endoplasmic reticulum [27].

### Compressive force

Compressive force plays an important role in osteoclastogenesis. Both compressive force and hypoxia may initiate osteoclastogenesis during OTM [28]. Heavy compression causes bone fracture of finger-like patterns [29]. TNF-α levels are higher on the compression side of periodontal ligament fibroblasts than on the tension side, which may influence RANKL expression during OTM [30]. Moreover, compression influences the osteodifferentiation of osteoblasts through the ClC-3 chloride channel in MC3T3-E1 cells, and regulates EphB4 and ephrinB2 expression [13,31]. Furthermore, nfatc-1, trap, rank, cath-K, clc7, mmp-9, atp6i, dc-stamp, and oc-stamp, and integrin-αv and -β3 are up-regulated by compression [32]. The effects of compression in OTM are dependent on the intensity of the force applied and are influenced by caffeine [33].

### Other forces

The application of high-frequency, low-intensity mechanical vibrations in the bone marrow mesenchymal stem cells of D1-ORL-UVA mice induces adipogenesis and alters their morphology [34]. Both photobiomodulation and low-amplitude high-frequency ultrasound enhance bone-fracture healing [35]. Low-intensity pulsed ultrasound (LIPUS) of appropriate strength and frequency significantly increases the bone tissue mineral density in mice. Thus, LIPUS may be clinically useful for maintaining bone integrity [36–38]. The effects of LIPUS involve the up-regulation of cyclooxygenase-2 and prostaglandin-E2, which are important in bone remodeling [39].

## A brief introduction to miRNAs

Classified as ssRNA molecules comprising an average of 22 nts, miRNAs inhibit gene expression at the DNA, RNA, and protein levels [40,41]. Almost 50% of miRNA genes are present in intergenic regions. Their expression is controlled by their own promoters, or in the case of polycistronic miRNA clusters, shared promoters [42]. In cancer [43], cardiovascular disease [41], COPD muscle dysfunction [44], osteoarthritis [45], and skin disorders [46], miRNAs play important roles as diagnostic biomarkers and molecular targetted therapies. miRNAs are regulated by, amongst others, TCF, β-catenin, and dickkopf-related protein 1 (Wnt signaling pathway) [47–50], and by Smad proteins (Smad7-Smad1/5/8-RUNX2 and Smad4-mediated pathways) [51–53]. miRNAs modulate the expression of histone deacetylase 4, forkhead box protein O1, Osterix (Osx), and growth/differentiation factor 5 during cell growth and differentiation [54–56].

Some reviews have addressed the role of miRNAs in bone remodeling and summarized the molecular pathways and specific proteins involved [57,58]. miRNAs function in the process of osteoporosis and bone resorption [58,59], indicating their therapeutic potential in tissue engineering in terms of interfering with bone resorption [60]. However, these reviews focussed on the effect of miRNAs on bone remodeling in the absence of mechanical forces. Therefore, we next discuss the role of miRNAs in bone formation induced by mechanical stimuli.

## miRNAs are involved in the mechanical force modulated bone metabolism

### MCS

miRNAs and their target genes form a complex network, the balance of which can be altered by mechanical forces; however, the underlying mechanism is unclear, despite the ever-increasing number of studies on the effects of MCS on bone metabolism. Most of these studies have used miRNA microarrays. The effects of changes in miRNA expression are mediated by proteins related to bone formation (e.g. RUNX2 and ALP). Overexpression of *miR-503-5p* in BMSCs attenuated stretch-induced osteogenic differentiation and decreased RUNX2 and ALP expression both *in vitro* and *in vivo* [61]. *miR-103-a*, which functions as an endogenous attenuator of RUNX2 in osteoblasts together with its target gene, *pank3*, was down-regulated during cyclical mechanical stretching-induced osteoblast differentiation, which led to an increase in RUNX2 protein levels [62]. Application of stretching force to periodontal ligament stem cells (PDLSCs) led to osteoblastic differentiation as well as changes in *miR-1246, miR-5096, miR-638, miR-663, miR-21, miR-4492*, and *miR-4734* expression. *miR-1246* is a novel target of p53 that can activate nuclear factor of activated T cells (NFAT) [63]. Moreover, activation of the NFAT pathway by RANKL attenuates osteoclast differentiation [64]. Under tensile stress, *miR-154-5p*, by targetting the Wnt/PCP pathway, prevents osteogenic differentiation of adipose-derived mesenchymal stem cells. *miR-195-5p*, which targets WNT3A, fibroblast growth factor 2 (FGF2), and bone morphogenetic protein receptor type IA (BMPR1A) [65] also inhibited osteogenic differentiation in PDLSCs. Indeed, inhibition of endogenous *miR-154-5p* using an antisense oligonucleotide significantly promoted osteogenic differentiation [66]. This may partly explain how mechanical stimulation activates the Wnt/β-catenin signaling pathway and promotes bone formation [67]. Exposure of PDLSCs to stretching force decreased the expression level of activin receptor type IIB (ACVR2B) via a direct interaction of *miR-21* with the 3′-untranslated repeat sequence of *ACVR2B* mRNA [68]. ACVR2B can regulate osteoblasts directly and negatively; therefore, *miR-21* plays a role in osteogenic differentiation [69]. The expression of *miR-138* in human bone marrow mesenchymal stem cells was decreased by mechanical tension. *miR-138* targets PTK2, which encodes focal adhesion kinase, a key mechanotransduction factor in osteogenesis. H19 may also be involved in this process [70].

Mechanical stretching down-regulates *miR-500, miR-1934, miR-31, miR-378*, and *miR-331* expression and up-regulates *miR-1941* expression by activating NF-κB in C2C12 myoblasts. Thus, miRNA expression during stretch-induced myoblast proliferation is dependent on NF-κB, which implies a mechanism for the intracellular transmission of external mechanical stimuli [71]. miRNAs are closely related to the components of regulatory networks involved in osteogenesis under mechanical stretching forces. *miR-33*, by acting through the *miR-33-BMP3-Smad* signaling pathway, prevents proliferation of venous SMCs in response to arterial stretching. *AgomiR-33* negatively regulates BMP3 expression and Smad2 and Smad5 phosphorylation [72]. BMP3, which inhibits Smad signaling and osteoblast differentiation, interacts with ACVR2B, as knockdown of endogenous ACVR2B in bone marrow stromal cells ameliorated the BMP3-mediated suppression of osteoblast differentiation [73,74]. In human trabecular meshwork cells, CMS induces the expression of *miR-24*, resulting in down-regulation of the subtilisin-like proprotein convertase Furin, which plays a major role in the processing of transforming growth factor β1 (TGF-β1). Down-regulation of Furin leads to weight loss; reduced bone mineral density (BMD), serum osteocalcin, total calcium, and intact parathyroid hormone levels; and an increased serum C telopeptide level. A similar finding was reported in osteoporotic postmenopausal females [75–77].

### FSS

Mechanical signals produced by FSS are sensed by mechanosensitive cells in bone and translated into biochemical signals [78]. This process is highly dependent on the sensitivity of cells to mechanical forces, which is closely associated with the chemistry of cell scaffolds on the surface [25]. It is thought that interstitial fluid flow and short-term FSS promote the terminal differentiation of pre-osteoblasts. Several miRNAs (*miR-20a, miR-21, miR-19b, miR-34a* and *-34c, miR-140*, and *miR-200b*) influence RUNX2 and ALP expression by targetting PPARy, Bambi, Crim l, TGFBR, SMAD3, PLAP-1, and TGFP1, which are key negative regulators of the BMP-SMAD-RUNX2-Osx signaling pathway [79]. Exposure to FSS significantly up-regulates *miR-132* expression in PDLCs. *miR-132* has regulatory effects through the PI3K/AKT/mTOR signaling pathway, as determined using the PI3K/AKT and mTOR signaling pathway inhibitor, BEZ235, which blocks FSS-induced differentiation in human PDL cells [80]. Wang et al. [81] reported that after FSS *miR-33-5p* and its target gene *Hmga2* negatively regulated osteoblast differentiation by modulating the proliferation of stem cells and MSCs [82]. Although few studies have focussed on the roles of miRNAs in the effects of FSS on bone formation, the signaling pathways and target genes involved have been identified. Further work should clarify the relationships between mechanical force and miRNA expression levels.

### Compressive force

Numerous studies have investigated the effects of compressive force on bone formation; however, few identified the underlying mechanism(s) in osteoblasts and osteoclasts. Iwawaki et al. [83] assessed miRNA expression during compressive treatment in MC3T3-E1 cells by microarray analysis. *miR-494-3p* was up-regulated after compression and inhibited the proliferation of MC3T3-E1 cells by modulating the mRNA levels of fibroblast growth factor receptor 2 (FGFR2) and Rho-associated coiled-coil kinase 1 (ROCK1). Both FGFR2 and ROCK1 are predicted to be targets of *miR-494-3p* and harbor *miR-494-3p* binding sites within the 3′-UTRs [83]. FGFR2 is a tyrosine kinase receptor involved in cell proliferation and differentiation [84]. Activation of the RhoA/ROCK pathway stimulates osteogenic and chondrogenic differentiation of mesenchymal stem cells [85]. The inhibition may be due to a reduced sealing zone area, which is an osteoclast-specific cytoskeletal structure that contributes to osteoclast-mediated bone resorption [86]. Consequently, compressive force inhibits osteoblast proliferation by up-regulating *miR-494-3p*, which suppresses FGFR2 and ROCK1 expression. Expression of *miR-29* in PDLCs is altered by compressive force, which affects the expression of several genes encoding major extracellular matrix (ECM) components negatively. In addition, Col1a1, Col3a1, and Col5a1 are direct targets of the *miR-29* family in PDLCs [87]. Indeed, Col3 reportedly plays a role in trabecular bone formation and maintenance by modulating osteogenesis [88]. In conclusion, compression regulates alveolar bone formation and ECM homeostasis in periodontal ligament. Further studies should focus on identifying the mechanism involved.

### Microgravity

The metabolism of osteoblasts is influenced by microgravity. Human mesenchymal stem cell (hMSC) differentiation into osteoblasts was suppressed in a ground-based, simulated microgravity environment, as indicated by the lack of expression of ALP, collagen 1, and osteonectin [89]. However, the function of miRNAs in microgravity-induced bone formation is unclear. *miR-132-3p* inhibits osteoblast differentiation and participates in the regulation of bone loss induced by simulated microgravity by targetting the gene encoding E1A-binding protein p300, a histone acetyltransferase important for the activity and stability of RUNX2 [90]. Moreover, *miR-33-5p* partially attenuates the microgravity-induced inhibition of MC3T3-E1 differentiation by targetting Hmga2; si targetting by *miR-495* prevents osteoblast proliferation and promotes apoptosis [81,91]. *miR-103* inhibits osteoblast proliferation by suppressing Cav1.2 expression in simulated microgravity [92,93].

## Discussion

**Figure 1:**
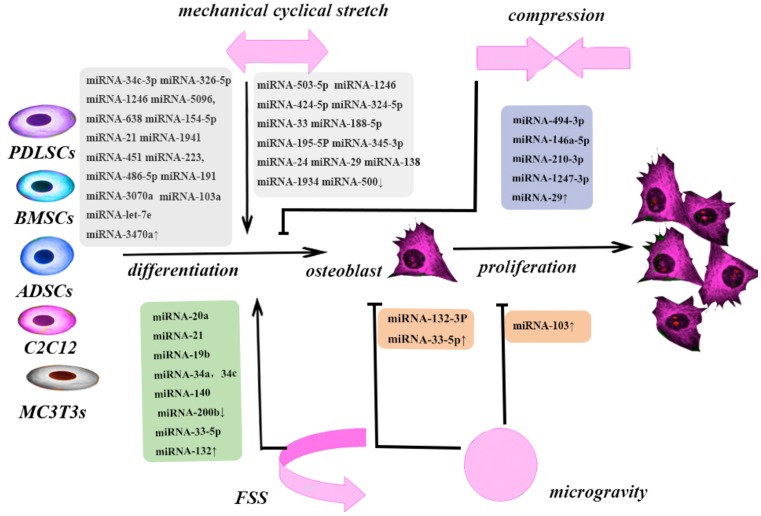
Different mechanical forces play different roles in bone remodeling

**Figure 2:**
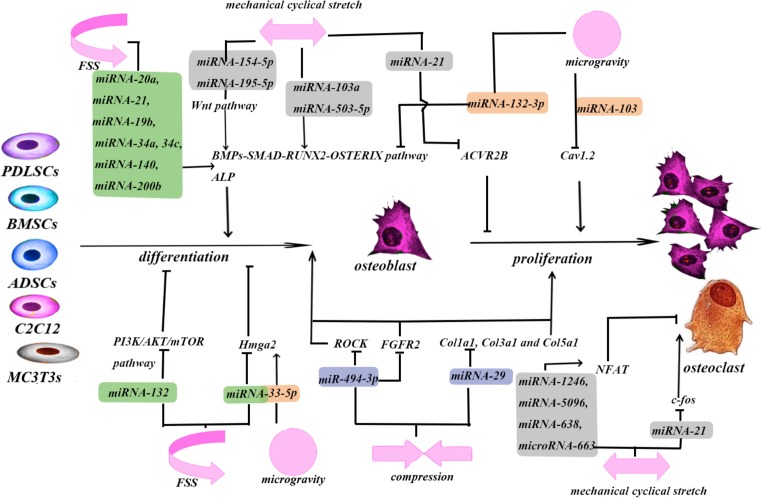
miRNAs mediate the process of bone remodeling as a post-transcriptional inhibitor

The relationship between mechanical force and miRNAs plays an important role in bone remodeling (Figure 1 and Table 1). The regulatory effects of miRNAs depend on a complex molecular network (Figure 2). miRNA microarrays are the most frequently used methods of detecting changes in miRNA expression. A mimic or inhibitor miRNA can be used to identify the target genes of candidate miRNAs. Dual-luciferase reporters may enable identification of combined sites of miRNAs on their target mRNAs. Wang et al. [81] assessed the relationship between *miR33-5p* and its target gene, *Hmga2*, by co-transfecting inhibitor-33 with siRNA-Hmga2. This resulted in partial blocking of the inhibitor-33-induced reduction in RUNX2, Osx, and ALP expression. Moreover, co-transfection of mimic-33 with pc-DNA3.1-Hmga2 or blank pcDNA3.1 also reduced the magnitude of the mimic-33-induced increases in RUNX2, Osx, and ALP expression [81]. Most studies on the effects of mechanical forces did not assess the effects of the intensity of the force applied. In animal studies, *miR-21* responded to orthodontic force in periodontal tissue in a dose- and time-dependent manner [5]. The levels of *miR195-5p* differed at 24, 48, and 72 h after application of tension force [65]. Therefore, the intensity of the mechanical force applied is also important. The optimum force can be different amongst types of stem cells. This could lead to clinical use of inhibitors of miRNAs.

**Table 1:** Studies about the role of miRNA in different forces induced bone metabolism

Mechanical force	miRNAs (express change with mechanical force)	Sample cells	Functions	Author
Compression	*miR-29*↑	PDLSCs and ABCs	Remodeling of alveolar bone (Col1a1, Col3a1, and Col5a1)	Chen [87]
Compression	*miR-494-3p, miR-146a-5p, miR-210-3p, miR-1247-3p* ↑	MC3T3-E2 cells	Osteoblast proliferation	Iwawaki [83]
FSS	*miR-20a miR-21 miR-19b miR-34a, 34c, miR-140 miR-200b*↓	MC3T3-E1 cells	Osteogenic differentiation	Mai [79]
FSS	*miR-132*↑	PDLSCs	Osteoblast differentiation and proliferation	Qi [80]
FSS	*miR-33-5p*↑	MC3T3-E1 cells	Osteoblast differentiation	Wang [81]
MCS	*miR-34c-3p miR-326-5p* ↑ *miR-503-5p, miR-324-5p, miR-188-5p, miR-345-3p, miR-30a-5p, miR-29b-3p, miR-351-3p*↓	RBMSCs	Osteogenic differentiation and bone formation.	Liu [61]
MCS	*miR-1246, miR-5096, miR-638, miR-663, miR-21, miR-4492, miR-4734*↑*miR-107, miR-423-5p* and *miR-3195*↓	PDLSCs	Osteoblast differentiation	Wei [119]
MCS	*miR-103a*↓	hFOB 1.19 cells	Osteoblast differentiation and bone formation	Zuo [62]
MCS	*miR-154-5p*↑	ADSC	Osteogenic differentiation of ADSCs	Li [66]
MCS	*miR-21*↑	PDLSCs	Osteogenic differentiation	Wei [68]
MCS	*miR-24*↓	HTM cell	Regulate the response to CMS	Luna [75]
MCS	*miR-29*↓	PDLSCs and ABCs	Remodeling of alveolar bone (Col1a1, Col3a1, and Col5a1)	Chen [87]
MCS	*miR-33*↓	SMCs	Cell proliferation	Huang [6]
MCS	*miR-500, miR-1934, miR-31, miR-378, miR-331*, and *miR-5097*↓ *miR-1941* ↑	C2C12 cells	Cell proliferation	Hua [71]
MCS	*miR-195-5p, miR-424-5p, miR-1297, miR-3607-5p miR-145-5p, miR-4328*, and *miR-224-5p*↓	PDLSCs	Bone formation	Chang [120]
MCS	*miR-195-5p*	PDLSCs	Osteogenic differentiation	Chang [65]
MCS	*miR-451, miR-223, miR-486-5p*↑ *miR-1246, miR-1260, miR-141*↓	PDLSCs	Periodontal tissue homeostasis	Stoecklin-Wasmer [121]
MCS	miR-191*, miR-3070a, miR-M1-2-3p miR-let-7e*, *miR-3470a*↑ *miR-32, miR-33, miR-5110*, and *miR-5121*↓	MC3T3-E1 cells	Osteoblast differentiation	Guo [122]
MCS	*miR-138*↓	hBMMSCs	Osteogenic differentiation	Wu [70]
Microgravity	*miR-103*↑	MC3T3-E1 cells	Osteoblast proliferation	Sun [92]
Microgravity	*miR-103*↑	MC3T3-E1 cells	Osteoblast proliferation	Sun [93]
Microgravity	*miR-132-3P*↑	MC3T3-E1 cells	Osteoblast differentiation	Hu [90]
Microgravity	*miR-33-5p*↓	MC3T3-E1 cells	Osteoblast differentiation	Wang [81]
Orthodontic force	*miR-21*↑	PDLSCs of mice	Osteogenesis of human PDLSCs following OTM	Chen [5]

Abbreviations: ABC, alveolar bone cell; hBMMSC, human marrow mesenchymal stem cell; HTM, human trabecular meshwork.

Irrespective of the amount of force used, MC3T3-E1 and PDLSC cells are the most frequently used *in vitro* studies of the effects of mechanical forces due to their function in osteogenesis. However, whether this occurs *in vivo* is unclear. *miR-21* expression in hPDLSE scraped from tooth’s roots is altered by application of orthodontic force for 1 month [5]. Rat models of OTM were used to assess the effects of stretching force on *miR-503-5p* and *miR-195-5p* expression [61,65]. Further *in vivo* studies are needed before miRNAs can be considered safe for clinical use.

Identifying the functions of miRNAs in the presence of mechanical force is problematic [94]. miRNAs play multiple roles in osteoblasts and osteoclasts in the presence of MCS, FSS, compressive force, and microgravity. miRNAs may regulate osteogenesis, which could be translated into novel therapeutic approaches for orthodontic conditions and bone fractures, as well as for systemic diseases, such as osteoporosis. miRNAs may be useful as diagnostic biomarkers and therapeutic agents [95]. The expression levels of miRNAs are significantly altered in fractured bone tissue; this is likely related to fracture healing. *miR-196a-3*, with its target gene *fgf2*, is associated with BMD [96]. *miR-145* attenuates TNF-α-driven cartilage matrix degradation in osteoarthritis by suppressing mitogen-activated protein kinase kinase 4 (MKK4) expression [97]. *miR-21, miR-22*, and *miR-30* regulate H_2_S production, which plays a role in bone formation as an osteoprotective factor [98]. In particular, *miR-21* regulates osteoblastogenesis and osteoclastogenesis to prevent bone resorption [99]. miRNAs can modulate the activity of bone formation-related factors and signaling pathways (e.g. BMPs and the NF-κB, RUNX2, Osx, and WNT signaling pathways). This facilitates the effects of other factors (e.g. Rorβ) on bone metabolism and enables these factors to function as regulators for treatment of bone-defect diseases [100–102]. Although few studies have evaluated the efficacy of miRNAs in orthodontics, miRNAs and miRNA inhibitors can be used clinically to regulate the tooth movement rate. The amount of secretory *miR-29* in gingival crevicular fluid was altered by orthodontic force, possibly due to the recruitment of osteoclasts [103]. This suggested the utility of adding specific miRNAs to gingival crevicular fluid during orthodontic treatment to change the tooth movement rate. Furthermore, use of exosomes as a tool for RNA transfer in therapeutic engineering has been reported [60], and exosomes from osteoclast precursors are involved in osteoclastogenesis [104]. Other biomaterials that mediated miRNA delivery, such as electrospun nanofiber scaffolds, chitosan, and CaP coated with PEGylated compounds, may be useful [105]. These technologies will lead to therapeutic applications of miRNAs as regulators of bone remodeling induced by orthodontic forces.

Mechanotransduction is critical for maintaining bone strength and quality under physiological conditions [106], and mechanical forces have been used to treat diseases involving bone loss. For instance, distraction osteogenesis is used to treat orthognathic and bone defects caused by trauma or a congenital cleft palate. Dentoalveolar distraction osteogenesis for canine retraction reportedly results in earlier tooth movement with minimal anchorage loss and a reduced treatment time compared with traditional distalization [107]; this has been confirmed in other studies [108–110]. Extracorporeal shockwave therapy is widely used in the treatment of bone-healing disturbances, vascular bone diseases, femoral hip osteonecrosis, and bone resorption in periodontal disease [111–114]. A previous study shed light on the relationship between mechanical force and bone metastasis in breast cancer [115]. Low intensity vibration (LIV) can increase bone density and can be used in the treatment of osteoporosis and fracture [116–117]. Moreover, mechanical loading is widely used to treat craniofacial deformities such as jaw discrepancies and cranial suture [118]. The above findings imply that modulating the expression levels of miRNAs could enhance the efficacy of mechanical force in the treatment of bone diseases

## Conclusion

The various effects of mechanical forces in bone remodeling are mediated by miRNAs as post-transcriptional regulators. Although the underlying mechanisms are unclear, previous studies suggest the therapeutic potential of miRNAs [95–105]. The antisense oligonucleotides of some miRNAs, such as anti-*miR-503*, anti-*miR-103*, anti-*miR-195*, may be used to promote the expression of RUNX2 and enhance osteoblast differentiation on the stretch side during orthodontic treatment. Other miRNAs, such as *miR-29*, may be used to promote osteoclast recruitment following compressive force. However, the dose and the time dependence of the effects of miRNAs should be investigated prior to their clinical application. Optimum vehicles for the transfer of miRNAs are also needed. Although miRNA-based orthodontic treatment is not yet available, this review may facilitate the development of novel miRNA-based therapies for orthodontics and for treatment of bone diseases such as osteoporosis and fracture.

## Abbreviations

ACVR2B: activin receptor type IIB
BMD: bone mineral density
circRNA: circular RNA
ECM: extracellular matrix
FGF2: fibroblast growth factor 2
FGFR2: fibroblast growth factor receptor 2
FSS: fluid shear stress
LIPUS: low-intensity pulsed ultrasound
lncRNA: long non-coding RNA
MCS: mechanical cyclical stretch
MSC: marrow mesenchymal stem cells
ncRNA: non-coding RNA
NFAT: nuclear factor of activated T-cell
OSX: Osterix
OTM: orthodontic tooth movement
PDL: periodontal ligament
PDLSC: periodontal ligament stem cell
RANKL: receptor activator of nuclear factor kappa-Β ligand
ROCK1: Rho-associated coiled-coil kinase 1
RUNX2: runt-related transcription factor 2
snoRNA: small non-translated nucleolar RNA
TRPV4: transient receptor potential cation channel subfamily V member 4
TNF-α: tumor necrosis factor alpha
